# Intra-athlete and inter-group comparisons: Running pace and step characteristics of elite athletes in the 400-m hurdles

**DOI:** 10.1371/journal.pone.0204185

**Published:** 2019-03-28

**Authors:** Mitsuo Otsuka, Tadao Isaka

**Affiliations:** Faculty of Sport and Health Science, Ritsumeikan University, Kusatsu, Shiga, Japan; The Ohio State University, UNITED STATES

## Abstract

The aim of this study was to investigate the running pace and step characteristics among various competitive-level 400-m hurdlers through inter-group and intra-athlete comparisons. We analysed spatiotemporal data involving the split time, mean step length (SL) and mean step frequency (SF) for 13 male world-class and 14 male national-level 400-m hurdlers. We analysed 16.5 ± 3.9 races for each world-class hurdler and 19.8 ± 6.0 races for each national-level hurdler (the total number of analysed runs was 491) using publicly available television and internet broadcasts. Inter-group comparisons showed that both first- and latter-halves split times of the world-class hurdlers were significantly shorter than those of the national-level hurdlers. In the latter-half phase, no significant differences of SL and SF were observed between the world-class and national level hurdlers. Intra-athlete comparisons showed that no athletes favoured only first-half phase in terms of running speed in short finish times. In contrast, finish times of all hurdlers were sensitive to running speed in the latter-half phase. In the latter half of the race, 18 of the 27 hurdlers were identified as being SF reliant during speed enhancements; running speed of the other 9 hurdlers were also sensitive to high SF. In conclusions, important findings regarding high performance in inter-group comparisons do not always corresponded with those in intra-athlete comparisons. All athletes and coaches should first prioritize maintaining high running speeds in the latter half of 400-m hurdles rather than in the first half of the race.

## Introduction

In a 400-m hurdles race for men, a total of ten hurdles at 91.4 cm height are positioned 35 m apart. So as to achieve and maintain high running speed during all inter-hurdle distances, it is essential for the 400-m hurdlers to enhance long sprint running ability [1], which is largely contributed by anaerobic glycolytic system [2–7]. In addition, hurdlers are needed to minimize decelerations in the running direction during clearing hurdles. Therefore, performance in 400-m hurdles is uniquely related to better techniques of adjusting step characteristics, unlike performance in 400-m flat runs.

Race pace is a key strategy for a good finish time in a long sprint running event [8]. As a significant number of previous studies reported notable changes in biochemical reactions after sprinting for 200-m during long sprint running events [4,6,8], the race paces are often investigated using a simple method of dividing the whole race into two parts [6,8]. During the hurdles race, split times are measured as touchdown split times, which are separated by the instant of touchdown of the leading leg [9]. Inter-group comparisons in 400-m hurdles races reported that the faster the finish times of hurdlers are, the faster touchdown split times are during the latter half of the race [10, 11]. However, because “correlation does not imply causation,” it is unclear whether the first- or latter-half split time is more important for each elite hurdler to improve the finish time. Nonetheless, to our knowledge, there are few intra-athlete comparisons on the running pace during the 400-m hurdles event.

Hierarchical models can help to prevent choosing performance arbitrarily and to give the necessary theoretical basis for examining the relative importance of various factors that affect the outcome a movement task [12]. Fast sprinting speed (short split time) is determined by high step frequency (SF) and high step length (SL); therefore, fast finish time can be hierarchically explained by relationships between split times and relationships between split times and step characteristics. SF and SL are often used to evaluate why some sprinters can run faster [13,14] and how sprinters can enhance running speed [15–17]. In 400-m hurdles race in the Olympic Games, most of hurdlers decrease the mean SL taken between hurdles from latter half of the race after clearing fifth hurdles [10]. When the mean SL between hurdles decreases, the running speed decreases [10]. Unfortunately, even previous studies on inter-group comparisons have not reported the detailed step characteristics of faster 400-m hurdlers in the first and latter halves of the race. The intra-athlete comparisons show that SF and/or SL reliance is based on individual changes to maintain high performance in a 100-m race [18] and long jump [19]. Hence, using a hierarchical model, it is required to investigate that high SF and/or high SL would achieve shortened split times and thereby a shortened finish time in the 400-m hurdles based on individual changes.

The influence of SL and SF on the 100-m finish time is individually different among various sprinters and the influence is not affected by the competitive level [18]. In contrast to the flat sprints, in the hurdles race, hurdlers are not often able to run with an optimal combination between SL and SF to achieve the highest running speed [20], which is externally caused by the fixed inter-hurdle distance [21]. In order to maintain the highest running speed as possible throughout the race, 400-m hurdlers are required to adjust step characteristics involving stride patterns in all fixed inter-hurdle distances, based on running fatigue. Higher competitive-level hurdlers would have the better techniques of adjusting step characteristics; therefore, effect of sensitivities of step characteristics on the split and finish times would be different between different competitive-level hurdlers.

Split time and step characteristics can be externally measured using a video camera [6,22]. In particular, researchers can easily analyse spatiotemporal parameters because they can calculate these parameters just by visually counting the number of steps in the running distance. Therefore, a number of samples can be obtained, for example, through public data analysis [18].

Both intra-athlete and inter-group comparisons would be helpful for individual athletes when determining the target race pace and step characteristics in the 400-m hurdles. Therefore, the purpose of this study was first, to investigate whether the hurdlers are individually more reliant on the first- or latter-half phase in terms of speed during the race and either having more SF or SL reliance during speed enhancements in each half of the race, and second, to investigate running pace and step characteristics between different competitive-level 400-m hurdlers cross-sectionally. We had three main hypotheses: First, the finish times of hurdlers would be individually influenced by their race pace and step characteristics. This hypothesis was hierarchically tested by intra-athlete comparisons across world-class and national-level hurdlers using public data analysis. Second, world-class 400-m hurdlers would run faster than national-level hurdlers in the latter half of the race. Third, the influences of step characteristics on the split time and finish time would differ between world-class and national-level hurdlers. Second and third hypotheses were tested comparing the two hurdler groups.

## Materials and methods

### Athletes

Because our data set was obtained from publicly-available Internet broadcasts, we did not obtain informed consent.

The sample comprised 13 male world-class and 14 male national-level 400-m hurdlers (best record: 47.71 ± 0.44 s and 49.28 ± 0.41 s, respectively). They were ranked in the top 20 more than three times from 2010 to 2014 in the World and Japan, respectively. This study was conducted after obtaining approval from the Research Ethics Committee involving Living Human Subjects at Ritsumeikan University (BKC-human-2016-053).

### Design

This study was laid out as an observational research design. We obtained the movie data during competitive races from publicly available television and internet broadcasts [18,23–25]. We conducted intra-athlete and inter-group comparisons to answer the research questions.

### Methodology

The competitions involved Olympic, International Association of Athletics Federation championships, Grand Prix series competitions, national championships, and local competitions in Japan (see, S1 Table). In this study, races in which the individual hurdler was likely to run with maximal effort through the finish line were analysed. We disregarded individual races in which the hurdler clearly slowed without maximal effort before the finish line from the analysis. A total of 16.5 ± 3.9 races (2012.4 ± 2.3 yr. race) for each world-class hurdler and 19.8 ± 6.0 races (2013.5 ± 1.6 yr. race) for each national-level hurdler were analysed; so the total number of the analysed runs was 491. The single mean values of finish times in all analysed trials were 48.84 ± 0.37 s for world-class hurdlers and 50.40 ± 0.43 s for national-level hurdlers, respectively. The research procedures complied with the companies' terms and conditions for all websites and broadcasts used in this study.

We used the finish time as the official race time. We determined the split times for the first and latter halves of the race at the instant of touchdown for the leading leg after clearing the fifth hurdle (hereafter, fifth touchdown).

We counted the total number of steps involved in clearing ten hurdles in the race until the first step over the finish line. During the first half of the race, we calculated the mean SL by dividing 185 m (from the start line to the position at the fifth hurdle) by the number of steps until the fifth touchdown and the mean SF by dividing the numbers of steps by the duration from the instant of the gunfire to the instant of the fifth touchdown. During the latter half of the race, we calculated the mean SL by dividing 215 m (from the position at the fifth hurdle to the finish line) by the number of steps to the finish time, and the mean SF by dividing the number of steps by the duration from the fifth touchdown to the finish line. Because most of the hurdlers did not complete a step exactly at 400 m, we estimated the total step number (*Step_total_*) using linear interpolation of the time prior to and after 400-m for each sprinter as follows:

$${Step}_{total}[step]={Step}_{Start-pre400}[step]+\frac{T_{pre400}}{T_{pre400}+T_{post400}}*1[step]$$

*Step*_*start*−400_ is the number of steps from start to just before the finish line. *T*_*pre*400_ is the duration from the touchdown of the step just before the finish line to the finish line. *T*_*post*400_ is the duration from the finish line to the touchdown of the step just after the finish line.

There were many trials analysed for each hurdler; therefore, single mean values and single standard deviation (SD) of split times, mean SF and mean SL in all analysed trials were calculated for each hurdler and utilized for further analysis.

### Statistical analysis

We present all parameters mean ± SD for two groups. We checked all datasets for normality and homogeneity of variance.

**Fig 1:**
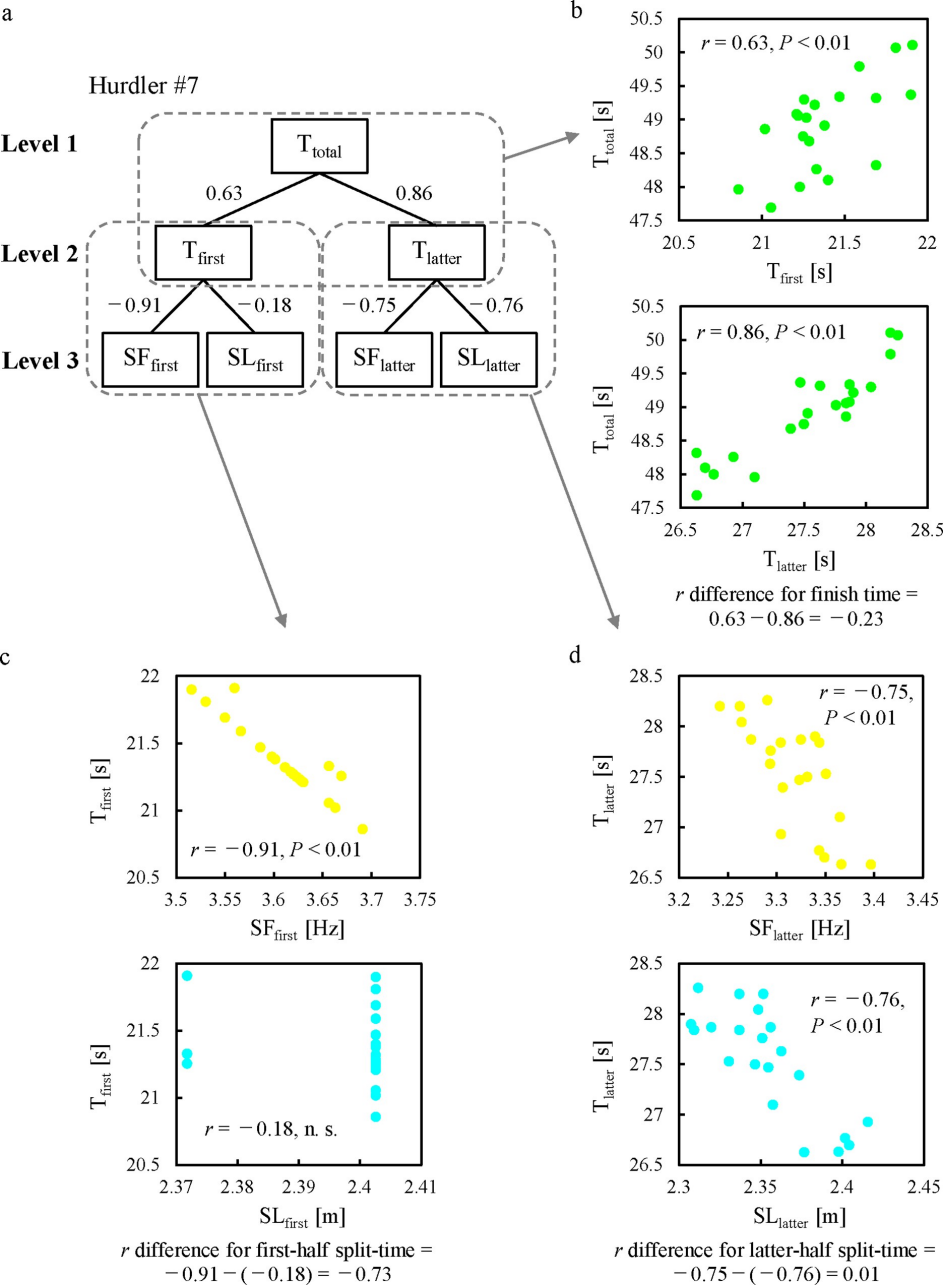
A typical example of intra-athlete relationships in terms of finish time, split time and step characteristics (hurdler #7). **(**a) A hierarchical relationship in terms of finish time (T_total_), split time (T_first_, first-half split lime; T_latter_, latter-half split time) and step characteristics (SF_first_, mean step frequency during the first half of the race; SL_first_, mean step length during the first half of the race; SF_latter_, mean step frequency during the latter half of the race; SL_latter_, mean step length during the latter half of the race) with three levels. The numbers show the correlation coefficients between two parameters which were natural log-transformed. (b) Scatter plots for T_total_, T_first_ and T_latter_. (c) Scatter plots for T_first_, SF_first_ and SL_first_. n. s. = not significant. (d) Scatter plots for T_latter_, SF_latter_ and SL_latter_. Points on the scatter-plot figures with trend lines are from original data whereas *r* values are from log-transformed data. The *r* difference for finish time and *r* differences for first- and latter-halves split times were calculated by the differences of the two correlation coefficients.

In the intra-athlete comparisons, we analysed each hurdler individually [8,16]. We natural log-transformed finish time, split time, mean SF, and mean SL before analysis to normalize distributions and stabilize variance. To determine any first- or latter-half speed reliance for an individual hurdler, we derived the 90% confidence interval (CI) for the difference between first-half split time vs. finish time and latter-half split time vs. finish time relationships (hereafter, *r* difference for finish time; Fig 1) using a criterion nonparametric bootstrapping technique (Matlab R2017a, Marthworks Inc., Natick, MA). Similarly, we calculated the 90% CI for the difference between the mean SL vs. split time and mean SF vs. split-time relationships in the first and latter halves of each race (hereafter, *r* difference for first-half split time and *r* difference for latter-half split time, respectively). We used Spearman’s correlations to calculate the intra-athlete relationships between the parameters for each resample. We employed a bootstrapping technique to provide 10,000 resamples of the natural log transformed finish time, split time, mean SL, and mean SF values. Salo et al. [18] present this data analysis in detail. We rigorously identified hurdlers as being first-half speed or SL reliant if the mean correlation difference was positive with the lower limit of the 90% CI ≥ +0.1; in contrast, we identified them as being latter-half speed reliant or SF reliant if the mean correlation difference was negative with the upper limit of the 90% CI ≤ −0.1.

To assess any differences were tested using paired and unpaired *T*-tests for parametric data. To assess any differences were tested using Wilcoxon and Mann-Whitney *U* tests were used. To assess the cross-sectional relationships between two parameters, we used Pearson’s correlations coefficients. Eta-squared was calculated for assessing the sample size in Wilcoxon and Mann-Whitney *U* tests.

We set statistical significance at *P* ≤ 0.05.

## Results

### Intra-athlete comparisons

A greater number of finish time−split time correlations were over 0.70 in the latter half of the race (24/27) compared to the first half (5/27) (Table 1). A greater number of correlations were less −0.70 in split time−mean SF compared to split time−mean SL in both first (21/27 vs. 0/27) and latter halves (19/27 vs. 4/27) of the race.

**Table 1:** Intra-athlete correlation coefficients among spatiotemporal parameters.

Hurdler	Level 1 & 2		Level 2 & 3			
	T_total_−T_first_	T_total_−T_latter_	T_first_−SF_first_	T_first_−SL_first_	T_latter_−SF_latter_	T_latter_−SL_latter_
**#1**	0.41	0.85	−1.00	0.00	−0.40	−0.40
**#2**	0.36	0.81	−1.00	0.00	−0.29	−0.66
**#3**	0.76	0.89	−0.29	−0.58	−0.61	−0.55
**#4**	0.35	0.89	−0.86	0.11	−0.80	−0.63
**#5**	0.67	0.97	−0.85	0.00	−1.00	−0.03
**#6**	0.45	0.65	−0.98	−0.15	−0.48	−0.89
**#7**	0.63	0.86	−0.91	−0.18	−0.75	−0.76
**#8**	0.74	0.74	−0.43	−0.68	−0.68	−0.19
**#9**	0.27	0.75	−0.97	0.15	−0.89	−0.50
**#10**	0.76	0.74	−0.98	−0.31	−0.84	−0.78
**#11**	0.52	0.67	−1.00	0.00	−0.80	−0.58
**#12**	0.35	0.97	−0.67	−0.10	−0.51	−0.57
**#13**	0.01	0.79	−1.00	0.00	−0.93	−0.06
**#14**	0.76	0.74	−0.87	−0.35	−0.70	−0.19
**#15**	0.35	0.77	0.29	−0.64	−0.82	−0.42
**#16**	0.68	0.79	−1.00	0.00	−0.76	−0.38
**#17**	0.63	0.64	−0.87	−0.06	−0.65	−0.50
**#18**	0.71	0.90	−0.97	−0.03	−0.94	−0.05
**#19**	0.53	0.70	−0.95	−0.24	−0.99	−0.23
**#20**	0.54	0.88	−1.00	0.38	−0.95	−0.71
**#21**	0.43	0.81	0.28	−0.31	−0.97	−0.34
**#22**	0.41	0.79	−0.58	−0.18	−0.88	−0.11
**#23**	0.63	0.86	−1.00	0.00	−0.84	−0.63
**#24**	0.33	0.84	−1.00	0.00	−0.78	−0.31
**#25**	0.60	0.89	−0.81	−0.36	−0.82	0.02
**#26**	0.16	0.82	−0.99	−0.39	−0.77	−0.42
**#27**	0.61	0.79	−1.00	0.00	−0.80	−0.11

Note. T_total_, finish time; T_first_, split time during the first-half phase; T_latter_, split time during the latter-half phase; SF_first_, mean step frequency during the first-half phase; SL_first_, mean step length during the first-half phase; SF_latter_, mean step frequency during the latter-half phase; SL_latter_, mean step length during the latter-half phase. The underlined numbers indicate that the magnitudes were over 0.70.

**Fig 2:**
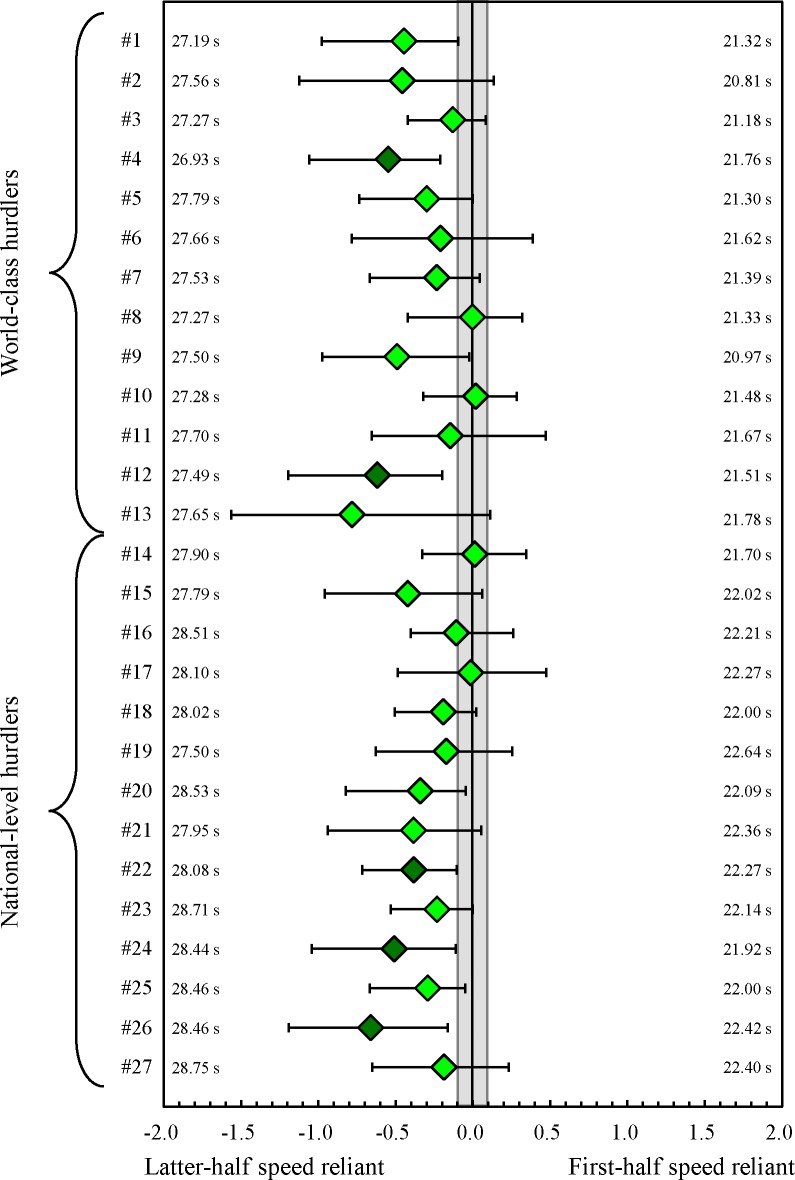
*r* difference for finish time (diamonds) with 90% confidence interval (bars) for each hurdler (#1 to #27). The area ±0.1 from zero in the middle demonstrates the trivial (nonreliant) effect. Green diamonds and the bar denote data from the latter-half speed reliant and yellow green diamonds and the bar denote data from the both first- and latter-halves speed reliant. Numbers on the right are single mean values of first-half split time in all trials. Numbers on the left are single mean values of latter-half split time in all trials.

**Fig 3:**
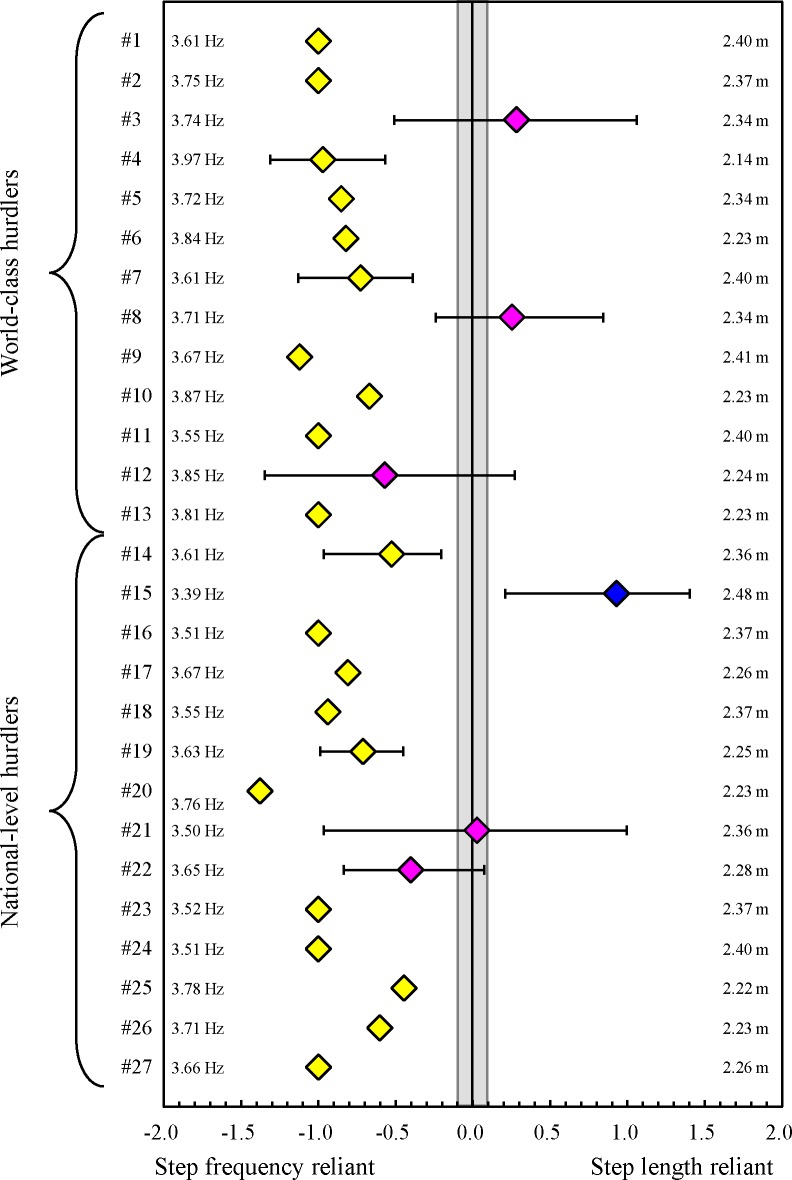
*r* difference for first-half split time with 90% confidence interval for each hurdler (#1 to #27). The area ±0.1 from zero in the middle demonstrates the trivial (nonreliant) effect. Yellow diamonds and the bar denote data from the step frequency reliant, blue diamonds and the bar denote data from the step length reliant and pink those denote data of the both step length and frequency reliant. Numbers on the right are single mean values of mean step length during the first-half phase in all trials. Numbers on the left are single mean values of mean step frequency during the first-half phase in all trials.

**Fig 4:**
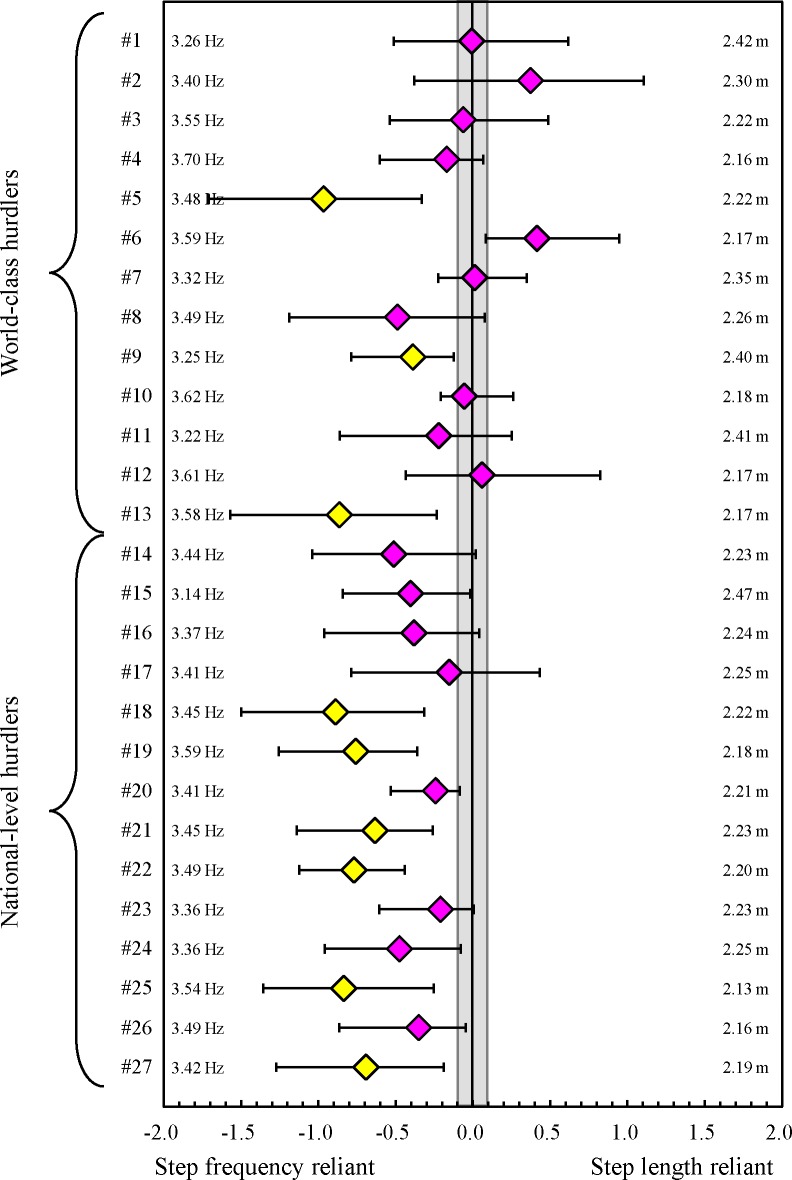
*r* difference for latter-half split time with 90% confidence interval for each hurdler (#1 to #27). The area ±0.1 from zero in the middle demonstrates the trivial (nonreliant) effect. Yellow diamonds and the bar denote data from the step frequency reliant and pink diamonds and the bar denote data from the both step length and frequency reliant. Numbers on the right are single mean values of mean step length during the latter-half phase in all trials. Numbers on the left are single mean values of mean step frequency during the latter-half phase in all trials.

5 of 27 athletes were identified as being latter-half speed reliant; in contrast, no athletes demonstrated first-half speed reliance and the 22 remaining athletes did not favour either characteristic (Fig 2). In the first half of the race, 21 of the 27 athletes were identified as being SF reliant, only one athlete demonstrated SL reliance, and the remaining five athletes did not favour either characteristic (Fig 3). In the latter half of the race, 9 of the 27 athletes were identified as being SF reliant, no athletes demonstrated SL reliance, and the remaining 18 athletes did not favour either characteristic (Fig 4). As the supporting information, intra-athlete relationships between split time and stride pattern, which is equal to the mean SL, are shown in S2 and S3 Tables.

### Inter-group comparisons

Single mean values of first- and latter-halves split times and finish time in all trials of the world-class hurdlers were significantly shorter than those of the top national-level hurdlers (Table 2). Single mean value of mean SF of the world-class hurdlers was significantly greater than compared to that of the national-level athletes during the first-half phase. In contrast, no significant difference of single mean value of mean SF and most frequent stride patterns (see S4 Table in detailed) were observed between two hurdler groups in each phase.

**Table 2:** Single mean value and SD of spatiotemporal parameters in all trials of world-class and national-level hurdlers (mean ± SD for two groups).

	World-class hurdlers	National- level hurdlers
**Single mean value**		
** T**_**total**_ **[s]**	48.84 ± 0.37 *	50.40 ± 0.43
** T**_**first**_ **[s]**	21.39 ± 0.29 *#§	22.17 ± 0.24 #§
** T**_**latter**_ **[s]**	27.45 ± 0.24 *	28.23 ± 0.38
** SF**_**total**_ **[Hz]**	3.59 ± 0.14	3.50 ± 0.10
** SF**_**first**_ **[Hz]**	3.75 ± 0.12 *#	3.60 ± 0.11 #
** SF**_**latter**_ **[Hz]**	3.47 ± 0.16	3.42 ± 0.11
** SL**_**total**_ **[m]**	2.29 ± 0.09	2.27 ± 0.07
** SL**_**first**_ **[m]**	2.31 ± 0.09 #	2.32 ± 0.08 #
** SL**_**latter**_ **[m]**	2.26 ± 0.10	2.23 ± 0.08
**Single SD**		
** T**_**total**_ **[s]**	0.60 ± 0.10	0.60 ± 0.11
** T**_**first**_ **[s]**	0.31 ± 0.07 #	0.31 ± 0.06 #
** T**_**latter**_ **[s]**	0.50 ± 0.08	0.50 ± 0.10
** SF**_**total**_ **[Hz]**	0.04 ± 0.01	0.04 ± 0.01
** SF**_**first**_ **[Hz]**	0.06 ± 0.01	0.05 ± 0.02 #
** SF**_**latter**_ **[Hz]**	0.05 ± 0.01	0.06 ± 0.01
** SL**_**total**_ **[m]**	0.02 ± 0.01	0.02 ± 0.01
** SL**_**first**_ **[m]**	0.01 ± 0.02 #	0.02 ± 0.02
** SL**_**latter**_ **[m]**	0.03 ± 0.02 *	0.02 ± 0.01

**Note.** A typical example for the calculation of single mean value and single SD is shown in S5 Table). T_total_ = finish time; T_first_ = split time during the first-half phase; T_latter_ = split time during the latter-half phase; SF_total_ = mean step frequency throughout the race; SF_first_ = mean step frequency during the first-half phase; SF_latter_ = mean step frequency during the latter-half phase; SL_total_ = mean step length throughout the race; SL_first_ = mean step length during the first-half phase; SL_latter_ = mean step length during the latter-half phase.

*Significant difference from national-level hurdlers (*P* < 0.05).

#Significant difference from latter-half phase (*P* < 0.05).

§When calculating the mean running speeds in each phase, significant difference was also obtained.

No significant differences in the single SDs for finish time, split time, and mean SF in all trials were obtained between the two different groups. However, the single SD for the latter-half mean SL of the world-class hurdlers was significantly larger than that of national-level hurdlers. As with the other parameters, the personal best times in the 400-m race of the world-class hurdlers were significantly shorter than those of the national-level hurdlers (45.74 ± 1.01 s vs. 47.42 ± 0.65 s, *P* < 0.01); in contrast, no significant difference in body height was observed between the two different groups (1.84 ± 0.07 m vs. 1.79 ± 0.05 m, n.s.).

**Fig 5:**
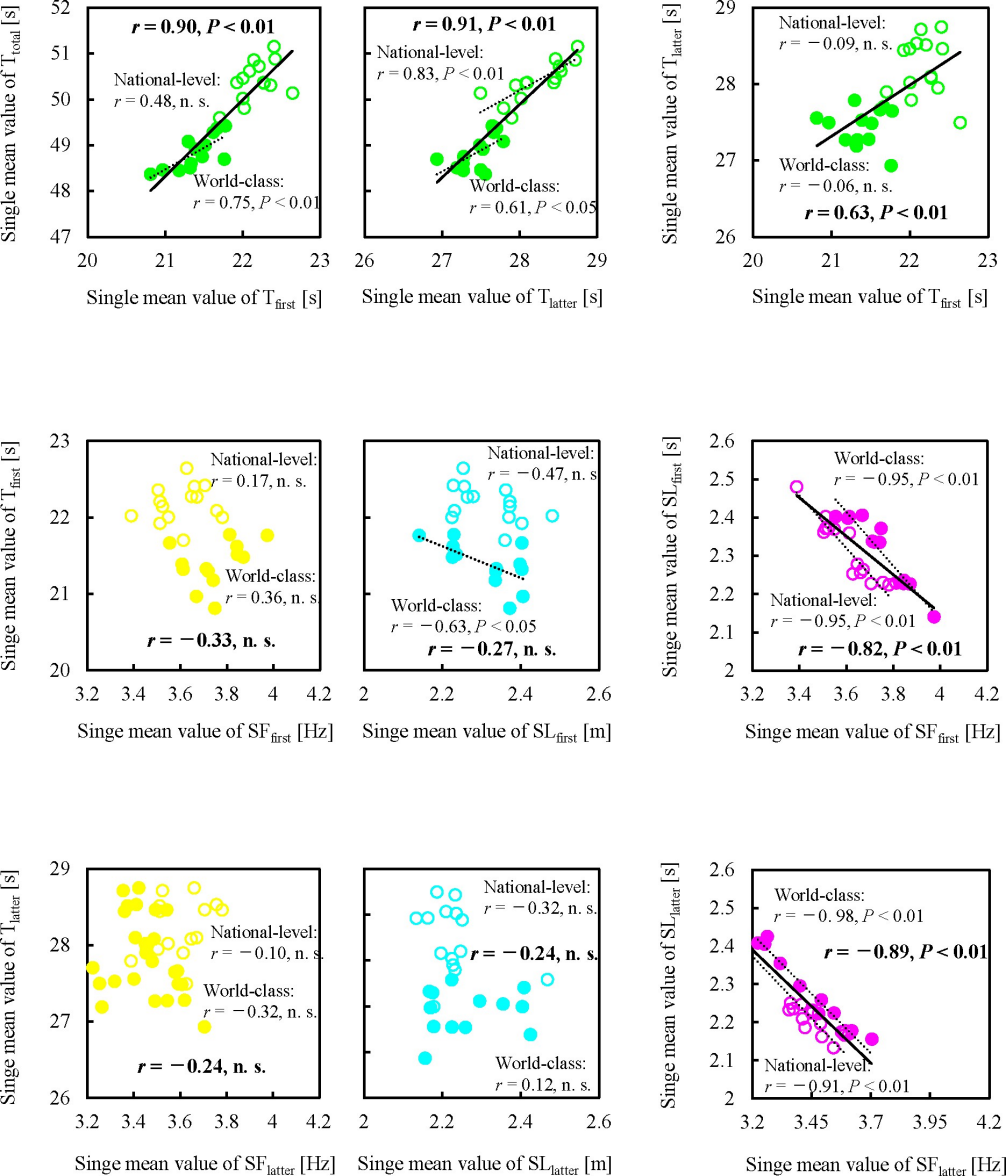
Scatter plots for single mean values of finish time, split time and step characteristics in inter-athlete relationships. The single mean value in all parameters were calculated from those in all trials (world-class hurdlers: 16.5 ± 3.9 races; national-level hurdlers: 19.8 ± 6.0 races). Filled circles denote data of world-class hurdlers and open circles denote data of national-level hurdlers. Solid lines indicate the regression lines for all hurdlers. Dot lines indicate the regression lines for world-class and national-level hurdlers separately. Single mean value of T_total_, single mean value of finish time; single mean value of T_first_, single mean value of split time during the first half of the race; single mean value of T_latter_, single mean value of split time during the latter half of the race; single mean value of SF_first_, single mean value of mean step frequency during the first half of the race; single mean value of SL_first_, single mean value of mean step length during the first half of the race; single mean value of SF_latter_, single mean value of mean step frequency during the latter half of the race; single mean value of SL_latter_, single mean value of mean step length during the latter half of the race; n. s., not significant.

Overall, positive relationships of finish times were significantly obtained between single mean values of first- and latter-halves split times (Fig 5). In contrast, no significant relationships of split-time were obtained between single mean values of mean SF and mean SL in either the first or latter half of the race. By dividing all hurdlers into two groups, in world-class hurdlers, a negative relationship was significantly observed between the single mean values of split time and mean SL during the first-half phase. Overall, the finish time of the 400-m hurdles was significantly related to personal records in the 400-m race (*r* = 0.73, *P* < 0.01); in contrast, by breaking two hurdler groups apart, only in world-class hurdlers, a positive relationship was significantly observed between them (world-class hurdlers: *r* = 0.54, *P* < 0.01; national-level hurdlers: *r* = −0.03, n. s.)

**Fig 6:**
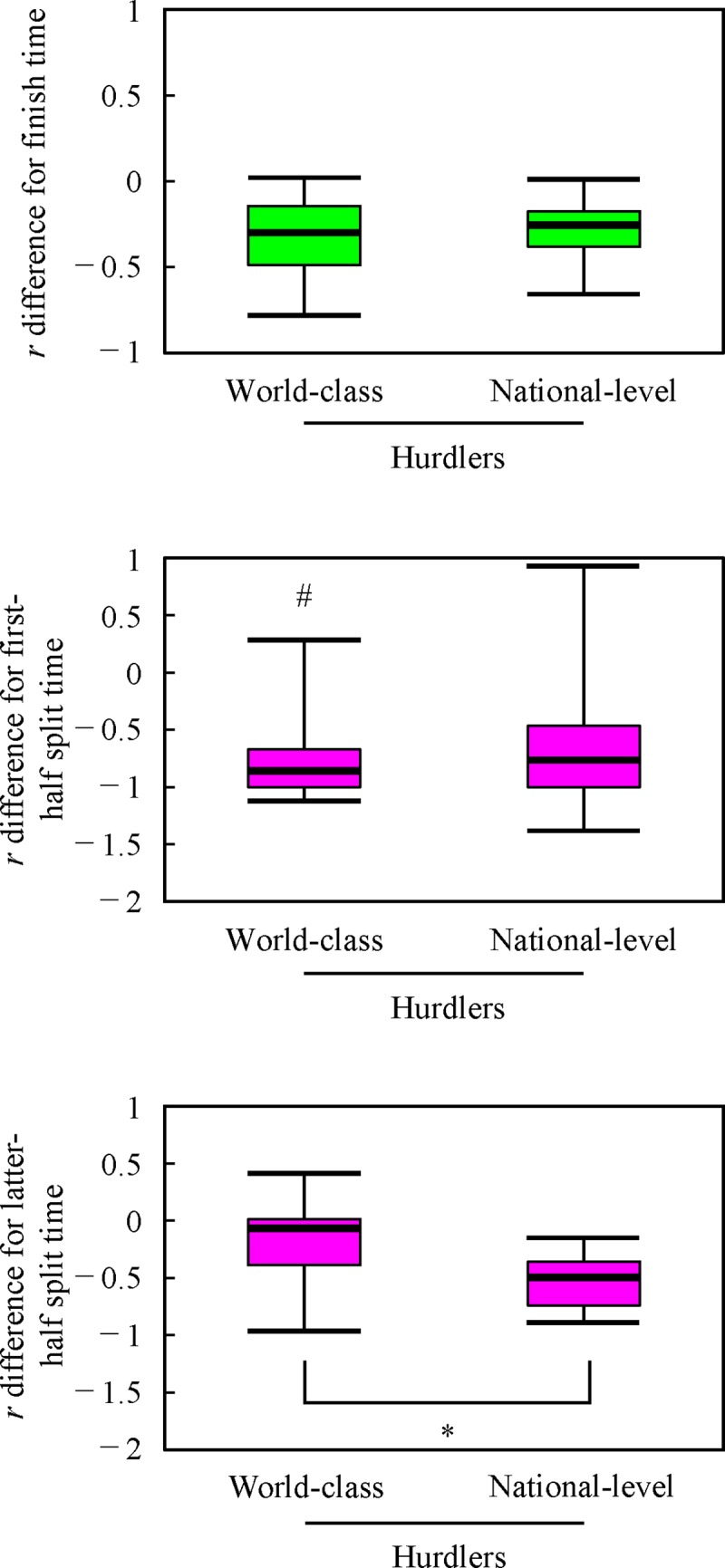
Box plot of the *r* differences for finish time, first- and latter-halves split times, comparing world-class and national-level hurdlers. The horizontal bars in the box plot indicate the highest value, upper quartile, median, lower quartile and lowest value from top to bottom, respectively. #Significant difference from latter-half phase (*P <* 0.05). *Significant difference between two groups (*P* < 0.05).

No significant differences in the *r* difference for finish time (*η*^2^ = 0.00) and the *r* difference for first-half split time (*η*^2^ = 0.02) were observed between world-class and national-level hurdlers (Fig 6). In contrast, the *r* difference for latter-half split time of the world-class hurdlers was significantly larger than that of national-level athletes (*η*^2^ = 0.35).

## Discussion

This study was the first to investigate the running paces and step characteristics of 400-m hurdlers using both intra-athlete and inter-group comparisons. Our main findings were as follows: first, even if the inter-group comparisons showed the extreme importance of the split time during both first and latter halves of the race, the intra-athlete comparisons showed that only latter-half split time was important for all hurdlers to better their finish times. Second, in the latter-half phase whose running speed was slower than that in the first-half phase, the step characteristics of world-class hurdlers were not different from the those of national-level hurdlers; in contrast, the mean SF was important for most of the hurdlers to decrease their split times. Third, the influence of mean SL on the split time in the latter half of the race was larger for the world-class hurdlers than for the national-level hurdlers. Therefore, the inter-group comparisons do not directly indicate causation in the 400-m hurdles, and the individualization principle for elite hurdler training can be considered essential.

In intra-athlete comparisons, the *r* difference for finish time is calculated by the difference between the two related correlation coefficients. Therefore, for the evaluation of the interrelationships, it is not possible to divide the whole race into three or four phases simultaneously. In this study, we simply divided the whole-race into first- and latter-halves phases, which led to a hierarchical relationship in terms of finish time, split time and step characteristics, in three levels as shown in Fig 1A. Great number of the finish time−split time correlations over 0.70 were obtained in the latter half of the race (27 of the 27 hurdlers). Interestingly, there were no first-half speed reliant hurdlers, who had only a high relationship between finish time and first-half split time, suggesting our first hypothesis was partially not accepted. These findings demonstrate that a shorter split time in the latter half of the race is more important for all hurdlers to achieve better personal finish times rather than a shorter split time in the first half of the race. Good split times in the latter half of a long sprint race are determined by several factors: small or optimal energy consumption in the first half of the race [6], increased utilization of glycolytic and aerobic systems [7], good technique while clearing the hurdles [26,27], and external environment such as wind (this effect changes depending on the combination of speed, direction, lane and timing of the blow motion) and high altitude [28]. Unfortunately, we cannot determine the factors in detail because of our observational methodology; however, these factors might affect the high performance of individuals in the latter half of the race.

In contrast to the intra-athlete comparisons, inter-group comparisons clarified that both single mean values of first- and latter-halves split times of the world-class hurdlers were significantly shorter than those of the national-level hurdlers. Overall, very strong inter-athlete relationships of single mean value of finish times were significantly observed between both single mean values of first- (*r* = 0.90) and latter-halves split times (*r* = 0.91). One previous study investigated the inter-athlete relationships of finish times between touchdown split times in all inter-hurdle distances and showed high relationships whose correlation coefficients were over 0.70 as observed in only the latter-half phase in the race [11]. These correlation coefficient values were lower as compared to those in this study. These differences in the strength of correlation coefficients, especially the relationship between the finish time and first-half split time, might be because the spatiotemporal data in this study were averaged from many competitive races for each hurdler (16.5 ± 3.9 races for world-class hurdlers and 19.8 ± 6.0 races for national-level hurdlers) so the acute daily effects of the sudden wind speed [28] and different race pace can be considered smaller compared to the analysis of only one race. In fact, the single SDs of the first- and latter-halves split times ranged widely over many competitive races (e.g., latter-half split time of world-class hurdlers: 0.50 ± 0.08 s). This finding suggests that analysing only one race for each hurdler cannot reflect the athlete’s normal performance; so, our cross-sectional methodology, which used the mean value for various races, can be considered as more accurate than that in a previous study [11]. Thus, our second hypothesis was accepted; furthermore, the high running speed in the first half of a race can be considered as an important factor in inter-group comparisons.

The intra-athlete comparisons clarified that, for most hurdlers, the short split time was more sensitive to high mean SF as compared to long mean SL during both first and latter halves of the race. At high sprinting speeds in short sprint running events, increases in step frequency have greater effects on increases in sprinting speeds [14,16,29]. This finding corresponds with our results. In contrast, our no significant inter-athlete relationships were observed between single mean values of split times and mean SF in both first and latter halves of the race. These suggest that important findings regarding high performance in inter-group comparisons do not always correspond with those in intra-athlete comparisons.

Moreover, we found that in world-class hurdlers, a significant negative relationship was observed between the single mean values of first-half split time and mean SL. In a 400-m race without hurdles, the differences in running speed among different level male sprinters are not caused by the differences in mean SF, but rather by the differences in mean SL throughout the whole race [8]. This finding supports our results. Conversely, in national-level hurdlers, no significant inter-athlete relationship was observed between the single mean values of split time and mean SL during the first-half phase. This might be due to the fact that a certain number of national-level hurdlers could not run with an optimal combination between mean SL and mean SF (over-lengthened SL with lower SF) to achieve the highest running speed [20] because they were forced to run as adjusting step characteristics in fixed inter-hurdle distances [21]. Indeed, even though the 400-m personal records of the national-level hurdlers were longer than those of the world-class hurdlers, which means they might have shorter SL during flat running [8], no significant difference in the single mean value of mean SL and pattern during the first-half phase was observed between the two groups.

In the latter half of the race, the mean SL is more sensitive to the split-time as compared to the during the first-half phase. The single SD of mean SL of the world-class hurdlers was significantly greater than that of the national-level hurdlers. Depending on the race, more world-class hurdlers (hurdler #1, #4, #6, #7 and #10 as shown in S3 Table) increased over two steps in the inter-hurdle distances and increased the split time as compared to national-level hurdlers (only hurdler #24 as shown in S3 Table). Therefore, even if the competitive level is higher in world-class hurdlers, the reliability of adjusting stride patterns is not higher compared to that in national-level hurdlers. This cannot be regarded as a good technique for the world-class hurdlers. The reason for the difference between two groups is unclear; however, this may lead the *r* difference for latter-half split time of the world-class hurdlers to be higher than that of the national-level hurdlers. Thus, our third hypothesis was accepted.

From the results in this study, we can provide the following information as practical applications for the 400-m hurdlers. First, when developing training programs and pace strategies, all hurdlers should prioritize improving high SF and running speed without shortening SL in the latter half of the 400-m hurdles rather than in the first half of the race. Second, improving performance in the first half of the race should be considered based on the individualization principle. This is because short finish times of all hurdlers were not sensitive to short first-half split times. Certain hurdlers should enhance their running speeds during the first-half phase in accordance with their physical conditions and/or motivation, other hurdlers should not enhance their running speeds even though their physical conditions and/or motivation are favourable. Evidence-based training will enhance running performance for 400-m hurdles [1].

We have three limitations in this study. First, we could not measure the parameters to assess biochemical energy expenditure. Long sprint running is performed by the glycolytic system with chemical energy supplies involving adenosine triphosphate and phosphocreatine, as well as aerobic systems [3–6,30]. This biochemical energy is finally converted to mechanical energy and various biomechanical parameters involving the step length and frequency [6,22], leading us to answer our question with a detailed explanation. Second, we did not measure the normal step length and step frequency during the 400-m flat race. Both world-class and national-level hurdlers may adjust the stride patterns before clearing hurdles in accordance with their levels of fatigue; however, we cannot confirm the manner in which they should adjust their stride patterns. Third, we did not investigate race paces of hurdlers with a more controlled experiment. The single SD of first-half split time of world-class hurdlers was 0.31 ± 0.07 s; however, the best racing strategy during the long sprint events is normally investigated with a wider range of running speeds during the first-half phase (e.g., the largest difference of 200-m split-time was 1.9 s [6]). However, during competition, it is very difficult to obtain these parameters. Further research is needed to answer our questions using parameters for biochemical energy expenditure, capacity for adjusting stride patterns and best racing strategy that would benefit individual athletes.

## Conclusion

In conclusion, although the inter-group comparisons showed running performances in the first and latter halves of the race of world-class hurdles were greater than that of national-level hurdlers, the intra-athlete comparisons showed latter-half performance is more essential for all hurdlers. Therefore, the important findings regarding high performance in inter-group comparisons do not always correspond with those in intra-athlete comparisons.

## Supporting information

S1 Table Summary of the analysed competitions. (XLSX)

S2 Table The most frequent stride-pattern during the first-half phase and parameters closely related to the split time by the stepwise regression analysis. (PDF)

S3 Table The most frequent stride pattern during the latter-half phase and parameters closely related to the split time by the stepwise regression analysis. (PDF)

S4 Table The most frequent number of steps in each running distance of world-class and national-level hurdlers. (PDF)

S5 Table Typical example for calculation of single mean value and single SD. (PDF)
