# Supplementary material for: Intra-athlete and inter-group comparisons: Running pace and step characteristics of elite athletes in the 400-m hurdles
Source: PLoS One. 2019 Mar 28;14(3):e0204185. doi: 10.1371/journal.pone.0204185 (PMC6438499; doi:10.1371/journal.pone.0204185)
Supplement: S2 Table — (PDF) [file pone.0204185.s002.pdf]

The most frequent stride pattern during the first-half phase and parameters closely related to the split time

To assess the relationships between the split time and several factors which associated with the stride pattern, stepwise multiple regression analyses were used. There were six factors considered during the first half phase as follows. 1) number of steps from start to take-off in the first hurdle clearance ($Step_{s\text{–}1}$), 2) the leading leg in the first hurdle clearance (1: left; 2: right), 3) number of steps between first and second hurdles ($Step_{1\text{–}2}$), 4) that between second to third hurdles ($Step_{2\text{–}3}$), 5) that between third and fourth hurdles ($Step_{3\text{–}4}$) and 6) that between fourth and fifth hurdles ($Step_{4\text{–}5}$).

S2 Table. The most frequent stride-pattern during the first-half phase and parameters closely related to the split time by the stepwise regression analysis

	Most frequent stride-pattern			Stepwise multiple analysis for first-half split time						
	$Step_{s\text{–}1}$-$Step_{1\text{–}2}$-$Step_{2\text{–}3}$-$step_{3\text{–}4}$-$step_{4\text{–}5}$	Leading-leg at 1st hurdle	Incidence [%]	Related parameters	*B*	*SEB*	*β*	*t*	*p*	Adjusted R^2
#1	20-13-13-13-13	Right	100	No parameters were selected.						
#2	21-13-13-13-13	Left	100	No parameters were selected.						
#3	21-13-13-13-13	Left	38							0.461
				$Step_{3\text{–}4}$ (13 or 14)	0.715	0.207	0.679	3.462	0.004	
#4	22-15-15-15-15	Left	86	No parameters were selected.						
#5	22-13-13-13-13	Right	90	No parameters were selected.						
#6	22-14-14-14-14	Left	94	No parameters were selected.						
#7	20-13-13-13-13	Left	82	No parameters were selected.						
#8	21-13-13-13-14	Left	39							0.259
				$Step_{1\text{–}2}$ (13 or 14)	0.447	0.169	0.550	0.264	0.018	
#9	20-13-13-13-13	Right	95	No parameters were selected.						
#10	22-14-14-14-14	Right	94	No parameters were selected.						
#11	20-13-13-13-13	Left	100	No parameters were selected.						
#12	22-14-14-14-14	Right	53	No parameters were selected.						
#13	22-14-14-14-14	Right	73	No parameters were selected.						

S2 Table. continued

	Most frequent stride-pattern			Stepwise multiple analysis for first-half split time						
	$Step_{s-1}$-$Step_{1-2}$-$Step_{2-3}$-$step_{3-4}$-$step_{4-5}$	Leading-leg at 1st hurdle	Incidence [%]	Related parameters	*B*	*SEB*	*β*	*t*	*p*	Adjusted R^2
#14	21-13-13-13-13	Right	60	No parameters were selected.						
#15	20-12-12-12-12	Right	48							0.375
				$Step_{3-4}$ (12 or 13)	0.364	0.101	0.637	3.605	0.002	
#16	21-13-13-13-13	Left	74	No parameters were selected.						
#17	21-14-14-14-14	Left	80	No parameters were selected.						
#18	21-13-13-13-13	Right	93	No parameters were selected.						
#19	21-14-14-14-14	Right	89	No parameters were selected.						
#20	22-14-14-14-14	Right	85							0.416
				$Step_{s-1}$ (21 or 22)	−1.084	0.284	−0.668	−3.812	0.001	
#21	21-13-13-13-13	Right	43							0.612
				$Step_{1-2}$ (13 or 14)	0.769	0.166	0.801	4.633	0.001	
#22	21-14-14-14-14	Right	44	No parameters were selected.						
#23	21-13-13-13-13	Left	100	No parameters were selected.						
#24	20-13-13-13-13	Right	100	No parameters were selected.						
#25	22-14-14-14-14	Right	95							0.477
				$Step_{4-5}$ (14 or 15)	0.838	0.187	0.708	4.486	0.000	
#26	22-14-14-14-14	Right	95	No parameters were selected.						
#27	21-14-14-14-14	Left	100	No parameters were selected.						

Underlined numbers in the most frequent stride-pattern indicate closely related parameters for the first-half split time. Shaded numbers and leading-leg indicate those changed based on races but did not closely related to the split time. Incidence indicates that of most frequent stride-pattern throughout all races in each hurdler. Numbers in parenthesis in the step wise multiple analysis indicate the step numbers that hurdlers used during the running distance. *B*, unstandardized regression coefficient; *SEB*, standard error of the unstandardized regression coefficient; *β*, standardized regression coefficient.
