# Supplementary material for: Intra-athlete and inter-group comparisons: Running pace and step characteristics of elite athletes in the 400-m hurdles
Source: PLoS One. 2019 Mar 28;14(3):e0204185. doi: 10.1371/journal.pone.0204185 (PMC6438499; doi:10.1371/journal.pone.0204185)
Supplement: S3 Table — (PDF) [file pone.0204185.s003.pdf]

The most frequent stride pattern during the latter-half phase and parameters closely related to the split time

To assess the relationships between the split time and several factors which associated with the stride pattern, stepwise multiple regression analyses were used. There were seven factors considered during the latter half phase as follows. 1) the leading-leg in the fifth hurdle clearance (1: left; 2: right), 2) number of steps between fifth and sixth hurdles ($Step_{5–6}$), 3) that between sixth and seventh hurdles ($Step_{6–7}$), 4) that between seventh and eighth hurdles ($Step_{7–8}$), 5) that between eighth and ninth hurdles ($Step_{8–9}$), 6) that between ninth and tenth hurdles ($Step_{9–10}$) and 7) that from landing the in tenth hurdle clearance to the finish line ($Step_{10–F}$).

S3 Table. The most frequent stride pattern during the latter-half phase and parameters closely related to the split time by the stepwise regression analysis

	Most frequent stride-pattern			Stepwise multiple analysis for latter-half split time						
	$Step_{5–6}$-$Step_{6–7}$-$Step_{7–8}$-$Step_{8–9}$-$Step_{9–10}$	Leading-leg at 5th hurdle	Incidence [%]	Related parameters	B	SEB	β	t	p	Adjusted R^2
#1	13-13-13-13-13	Right	40							0.332
				$Step_{9–10}$ (13 or 15)	0.346	0.107	0.606	3.232	0.005	
#2	13-14-14-15-15	Left	31							0.629
				$Step_{10-F}$ (17.3–19.1)	0.880	0.204	0.793	4.316	0.001	
#3	14-14-15-15-15	Left	56							0.451
				$Step_{10-F}$ (18.0–19.3)	0.931	0.255	0.698	3.650	0.003	
#4	15-15-15-15-15	Left	73							0.330
				$Step_{9–10}$ (15, 16 or 17)	0.356	0.106	0.601	3.367	0.003	
#5	14-14-15-15-15	Right	100	No parameters were selected.						
#6	14-15-15-15-15	Left	41							0.698
				$Step_{9–10}$ (15 or 16)	0.736	0.136	0.751	5.422	0.000	
				$Step_{5–6}$ (14 or 15)	0.424	0.178	0.330	2.386	0.032	
#7	13-13-14-14-14	Left	18							0.536
				$Step_{9–10}$ (14 or 15)	0.636	0.165	0.589	3.859	0.001	
				$Step_{7–8}$ (13 or 14)	0.522	0.177	0.450	2.951	0.009	
#8	14-14-14-15-15	Left	28							0.209
				$Step_{6–7}$ (14 or 15)	0.529	0.226	0.506	2.344	0.032	
#9	13-13-13-14-14	Right	90	No parameters were selected.						
#10	14-15-15-15-15	Right	41							0.734
				$Step_{9–10}$ (15 or 16)	0.544	0.109	0.696	5.008	0.000	
				$Step_{5–6}$ (14 or 15)	0.738	0.136	0.754	5.434	0.000	
				$Step_{10–F}$ (18.3–19.8)	0.309	0.134	0.308	2.305	0.038	
#11	13-13-13-14-14	Left	69	No parameters were selected.						
#12	14-15-15-15-15	Right	33							0.437
				$Step_{9–10}$ (15 or 16)	0.587	0.170	0.691	3.445	0.004	
#13	14-15-15-15-15	Right	64	No parameters were selected.						

Table S3. continued

	Most frequent stride-pattern $Step_{5-6}$-$Step_{6-7}$-$Step_{7-8}$-$Step_{8-9}$-$Step_{9-10}$	Leading-leg at 5th hurdle	Incidence [%]	Stepwise multiple analysis for latter-half split time: Related parameters	*B*	*SEB*	*β*	*t*	*p*	Adjusted R^2
#14	14-14-15-15-15	Right	55	No parameters were selected.						
#15	13-13-13-13-13	Right	81	No parameters were selected.						
#16	14-15-15-15-15	Left	32	$Step_{6-7}$ (14 or 15)						0.208
					0.437	0.183	0.502	2.393	0.029	
#17	14-14-14-14-15	Left	40	$Step_{10-F}$ (17.8–19.3)						0.288
					0.673	0.265	0.576	2.537	0.025	
#18	14-14-15-15-15	Right	93	No parameters were selected.						
#19	15-15-15-15-15	Right	100	No parameters were selected.						
#20	14-14-15-15-15	Right	55							0.513
				$Step_{9-10}$ (15 or 16)	1.144	0.249	0.734	4.588	0.000	
#21	14-14-15-15-15	Right	64	No parameters were selected.						
#22	14-15-15-15-15	Right	51	No parameters were selected.						
#23	13-15-15-15-15	Left	74							0.489
				$Step_{10-F}$ (17.7–19.2)	0.965	0.226	0.719	4.267	0.001	
#24	14-14-15-15-15	Right	32							0.402
				$Step_{9-10}$ (15 or 16)	0.962	0.330	0.534	2.921	0.010	
				$Step_{8-9}$ (14, 15 or 16)	0.396	0.183	0.395	2.161	0.046	
#25	15-15-15-16-16	Right	59	No parameters were selected.						
#26	14-15-15-15-15	Right	42							0.384
				$Step_{8-9}$ (15 or 16)	0.667	0.205	0.620	3.257	0.005	
#27	14-14-15-15-15	Left	50							0.457
				$Step_{9-10}$ (15 or 16)	1.400	0.358	0.699	3.913	0.001	

Underlined numbers in the most frequent stride-pattern indicate closely related parameters for the first-half split time. Shaded numbers and leading-leg indicate those changed based on races but did not closely related to the split time. Incidence indicates that of most frequent stride-pattern throughout all races in each hurdler. Numbers in parenthesis in the step wise multiple analysis indicate the step numbers that hurdlers used during the running distance. *B*, unstandardized regression coefficient; *SEB*, standard error of the unstandardized regression coefficient; *β*, standardized regression coefficient.
