# Supplementary material for: Intra-athlete and inter-group comparisons: Running pace and step characteristics of elite athletes in the 400-m hurdles
Source: PLoS One. 2019 Mar 28;14(3):e0204185. doi: 10.1371/journal.pone.0204185 (PMC6438499; doi:10.1371/journal.pone.0204185)
Supplement: S4 Table — (PDF) [file pone.0204185.s004.pdf]

The most frequent number of steps in each running distance of world-class and national-level hurdlers.

To assess the differences of the number of steps in each running distance between two groups, Mann-Whitney *U* tests were used. The number of steps in each running distance was selected using the most frequent that in each half phase in all trials.

S4 Table. The most frequent number of steps in each running distance of world-class and national-level hurdlers

	N (%)		Mann-Whitney *U* test	
	World-class hurdlers	National-level hurdlers	*P*	η^2
$Step_{s\text{-}1}$			n. s.	0.01
20 steps	4 (31%)	2 (14%)		
21 steps	3 (23%)	9 (64%)		
22 steps	6 (46%)	3 (21%)		
$Step_{1\text{-}2}$			n. s.	0.00
12 steps	0 (0%)	1 (7%)		
13 steps	8 (62%)	6 (43%)		
14 steps	4 (31%)	7 (50%)		
15 steps	1 (8%)	0 (0%)		
$Step_{2\text{-}3}$			n. s.	0.00
12 steps	0 (0%)	1 (7%)		
13 steps	8 (62%)	6 (43%)		
14 steps	4 (31%)	7 (50%)		
15 steps	1 (8%)	0 (0%)		
$Step_{3\text{-}4}$			n. s.	0.00
12 steps	0 (0%)	1 (7%)		
13 steps	8 (62%)	6 (43%)		
14 steps	4 (31%)	7 (50%)		
15 steps	1 (8%)	0 (0%)		
$Step_{4\text{-}5}$			n. s.	0.01
12 steps	0 (0%)	1 (7%)		
13 steps	7 (54%)	6 (43%)		
14 steps	5 (38%)	7 (50%)		
15 steps	1 (8%)	0 (0%)		
$Step_{5\text{-}6}$			n. s.	0.07
13 steps	5 (38%)	2 (14%)		
14 steps	7 (54%)	10 (71%)		
15 steps	1 (8%)	2 (14%)		
$Step_{6\text{-}7}$			n. s.	0.03
13 steps	4 31%)	1 (7%)		
14 steps	4 (31%)	7 (50%)		
15 steps	5 (38%)	6 (43%)		
$Step_{7\text{-}8}$			n. s.	0.11
13 steps	3 (23%)	1 (7%)		
14 steps	3 (23%)	1 (7%)		
15 steps	7 (54%)	12 (86%)		
$Step_{8\text{-}9}$			n. s.	0.05
13 steps	1 (8%)	1 (7%)		
14 steps	3 (23%)	1 (7%)		
15 steps	9 (69%)	11 (79%)		
16 steps	0 (0%)	1 (7%)		
$Step_{9\text{-}10}$			n. s.	0.10
13 steps	1 (8%)	1 (7%)		
14 steps	3 (23%)	0 (0%)		
15 steps	9 (69%)	12 (86%)		
16 steps	0 (0%)	1 (7%)		

$Step_{s–1}$, number of steps from start to take-off in the first hurdle clearance; $Step_{1–2}$, number of steps between first and second hurdles; $Step_{2–3}$, number of steps between second to third hurdles; $Step_{3–4}$, number of steps between third and fourth hurdles; $Step_{4–5}$, between fourth and fifth hurdles; $Step_{5–6}$, number of steps between fifth and sixth hurdles; $Step_{6–7}$, number of steps between sixth and seventh hurdles; $Step_{7–8}$, number of steps between seventh and eighth hurdles; $Step_{8–9}$, number of steps between eighth and ninth hurdles; $Step_{9–10}$, number of steps between ninth and tenth hurdles.
