# Supplementary material for: Intra-athlete and inter-group comparisons: Running pace and step characteristics of elite athletes in the 400-m hurdles
Source: PLoS One. 2019 Mar 28;14(3):e0204185. doi: 10.1371/journal.pone.0204185 (PMC6438499; doi:10.1371/journal.pone.0204185)
Supplement: S5 Table — (PDF) [file pone.0204185.s005.pdf]

Typical example for calculation of single mean value and single SD

Because there were many analysed races for one hurdler (world-class hurdlers: 16.5 ± 3.9 races; national-level hurdlers: 19.8 ± 6.0 races), mean value and SD in all parameters were initially calculated for one hurdler (these are single mean value and single SD). Next, mean value ± SD were calculated in each single mean value and single SD in all parameters from many different hurdlers (n = 27). Typical example for calculation of single mean value and single SD in finish time of world-class hurdlers is shown in S5 Table.

S5 Table Typical example for calculation of single mean value and single SD in finish time of world-class hurdlers

Subject	Raw data of finish time [s]	n	Single mean value	Single SD
#1	47.56, 47.61, 47.86, 47.91, 48.11, 48.12, 48.12, 48.17, 48.21, 48.33, 48.36, 48.50, 48.74, 48.75, 48.85, 48.89, 48.93, 49.08, 49.48, 50.63	20	48.51	0.71
#2	47.25, 47.50, 47.95, 47.97, 48.25, 48.29, 48.43, 48.53, 48.57, 48.86, 48.91, 48.98, 49.31	13	48.37	0.59
#3	47.25, 47.63, 47.63, 47.76, 48.01, 48.10, 48.19, 48.22, 48.56, 48.80, 48.87, 49.00, 49.01, 49.21, 49.31, 49.68	16	48.45	0.70
#4	47.65, 47.94, 48.09, 48.22, 48.23, 48.23, 48.25, 48.25, 48.53, 48.55, 48.67, 48.71, 48.81, 48.92, 48.94, 48.97, 49.11, 49.11, 49.12, 49.24, 49.62, 50.14	22	48.70	0.58
#5	48.13, 48.20, 48.31, 48.67, 48.97, 49.13, 49.52, 49.71, 50.04, 50.17	10	49.09	0.76
#6	48.63, 48.72, 48.74, 48.80, 48.88, 48.92, 48.96, 48.97, 48.98, 49.05, 49.27, 49.52, 49.79, 49.86, 50.05, 50.31, 50.38	17	49.28	0.58
#7	47.69, 47.96, 48.00 48.10, 48.26, 48.32, 48.68, 48.75, 48.86, 48.91, 49.03, 49.06, 49.08, 49.22, 49.30, 49.32, 49.34, 49.37, 49.79, 50.07, 50.11	21	48.92	0.68
#8	47.70, 47.91, 47.98, 48.18, 48.31, 48.31, 48.33, 48.43, 48.47, 48.60, 48.79, 48.91, 48.92, 48.97, 49.02, 49.03, 49.07, 49.86	18	48.60	0.52
#9	47.78, 47.78, 47.93, 48.09, 48.10, 48.32, 48.35, 48.36, 48.38, 48.42, 48.44, 48.47, 48.50, 48.50, 48.52, 48.53, 48.60, 48.66, 48.74, 49.36, 49.91	21	48.46	0.48
#10	47.84, 48.10, 48.19, 48.24, 48.26, 48.43, 48.52, 48.52, 48.62, 48.68, 48.78, 48.81, 49.25, 49.47, 49.53, 49.79, 49.85	17	48.76	0.61
#11	48.13, 48.56, 48.81, 49.18, 49.33, 49.33, 49.43, 49.47, 49.53, 49.63, 49.64, 49.93, 50.85	13	49.37	0.66
#12	48.25, 48.34, 48.50, 48.52, 48.78, 48.83, 48.85, 48.96, 49.12, 49.18, 49.20, 49.31, 49.35, 49.66, 50.15	15	49.00	0.51
#13	48.96, 48.96, 48.97, 49.09, 49.12, 49.29, 49.63, 49.63, 49.85, 50.02, 50.13	11	49.42	0.44
Mean value		16.5	48.84	0.60
SD		3.9	0.37	0.10
